# The James Lind Initiative: books, websites and databases to promote critical thinking about treatment claims, 2003 to 2018

**DOI:** 10.1186/s40900-019-0138-2

**Published:** 2019-02-04

**Authors:** Iain Chalmers, Patricia Atkinson, Douglas Badenoch, Paul Glasziou, Astrid Austvoll-Dahlgren, Andy Oxman, Mike Clarke

**Affiliations:** 1 James Lind Initiative, Oxford, OX2 7LG UK; 2 Minervation Ltd, The Wheelhouse, First Floor, Angel Court, 81 St Clements Street Oxford, England, OX4 1AW UK; 3 Faculty of Health Sciences and Medicine, Bond University, Gold Coast, QLD 4229 Australia; 4 Regional Centre for Child and Adolescent Mental Health, Eastern and Southern Norway, Gullhaugveien 1-3, 0484 Oslo, Norway; 5 Centre for Informed Health Choices, Norwegian Institute of Public Health, Box 4404, Nydalen, N-0403 Oslo, PO Norway; 6 Centre for Public Health, Institute of Clinical Sciences, Block B, Queens University Belfast, Royal Hospitals, Grosvenor Road, Belfast, BT12 6BJ UK

**Keywords:** James Lind library, James Lind Alliance, Testing treatments, Testing treatments interactive, Testing treatments international, Key concepts, Informed health choices projects, Claim evaluation tools, Critical thinking and appraisal resource library (CARL), GET-IT glossary, Teachers of EBHC, GenerationR, Evaluation of teaching/learning

## Abstract

**Background:**

The James Lind Initiative (JLI) was a work programme inaugurated by Iain Chalmers and Patricia Atkinson to press for better research for better health care. It ran between 2003 and 2018, when Iain Chalmers retired. During the 15 years of its existence, the JLI developed three strands of work in collaboration with the authors of this paper, and with others.

**Work themes:**

The first work strand involved developing a process for use by patients, carers and clinicians to identify shared priorities for research – the James Lind Alliance. The second strand was a series of articles, meetings, prizes and other developments to raise awareness of the massive amounts of avoidable waste in research, and of ways of reducing it. The third strand involved using a variety of approaches to promote better public and professional understanding of the importance of research in clinical practice and public health. JLI work on the first two themes has been addressed in previously published reports. This paper summarises JLI involvement during the 15 years of its existence in giving talks, convening workshops, writing books, and creating websites and databases to promote critical thinking about treatment claims.

**Conclusion:**

During its 15-year life, the James Lind Initiative worked collaboratively with others to create free teaching and learning resources to help children and adults learn how to recognise untrustworthy claims about the effects of treatments. These resources have been translated in more than twenty languages, but much more could be done to support their uptake and wider use.

## Plain English summary

We are bombarded with claims about the effects of treatments. These reach us through advertising, broadcasts, newspapers, health professionals, family, and friends. People often trust these sources of information more than they trust the results of research. Because claims about the effects of treatments can be wrong, including those based on poor research, accepting them uncritically can result in harm. This is why people need to be equipped to spot untrustworthy treatment claims.

The skills needed to identify dodgy treatment claims are not often elements of people’s general knowledge. Between 2003 and 2018, in collaboration with others, the James Lind Initiative worked to promote an increase in the general knowledge needed to identify untrustworthy treatment claims, and so promote better research for better health care. This entailed giving talks, convening workshops, writing books, and creating websites and databases to promote critical thinking about treatment claims – among children as well as adults. This article describes the origins and evolution of these initiatives, and the free teaching and learning resources that have resulted.

## The James Lind Initiative

In 2003, following acceptance of the report of a UK Medical Research Council (MRC) working party entitled ‘Clinical Trials for Tomorrow’, the Council declared its commitment to ‘involve patients in all aspects of the clinical trials it funds’. Iain Chalmers and Patricia Atkinson were appointed by the MRC and the Department of Health to establish:

*a communications and discussion forum on randomised controlled trials, involving patients, practitioners, researchers and others.*

Outline plans for an initiative responding to this brief were introduced by Iain Chalmers in an article published in the *Journal of the Royal Society of Medicine* at the end of 2003. The article noted that the initiative was being established “to lobby for better randomized controlled trials because these studies can provide some of the most important information needed to improve healthcare.” It went on to note that:

*Various strategies are likely to be needed if controlled trials are to improve. One involves promoting greater public demand for better, more relevant controlled trials. Because of the various perverse incentives that distort the research agenda, patients and their representatives should be encouraged to discriminate between those controlled trials that deserve their support and those that do not. Involvement of people using the health services in all phases of controlled trials should help to ensure that these studies address issues that are of real importance, and that the results are made publicly available. In particular, patients and the public need to press for funding of trials addressing questions that are of importance to patients but of no interest to industry (Chalmers 2003)* [1]*.*

The initiative was named for James Lind, the eighteenth century Scottish naval surgeon, who organised a controlled trial to resolve uncertainty about how to treat scurvy (Lind 1753) [2].

Most of the subsequent work of the James Lind Initiative (JLI), fell within one of three main strands, all of which were concerned with engaging with patients, professionals and the public. The first strand involved the piloting and development of the James Lind Alliance (JLA), an interactive process to help patients, carers and clinicians to identify shared research priorities (Chalmers et al. 2013) [3]. This work exposed a substantial mismatch between the interventions that patients and clinicians wished to see evaluated and those (mainly drugs) that were being assessed by researchers (Crowe et al. 2015). [4] After the JLI’s development of the JLA Priority Setting Partnerships, responsibility for additional applications and development passed to the National Institute for Health Research in 2013 for maintenance and further development (http://www.jla.nihr.ac.uk/).

A second strand of the JLI’s work - to expose and confront avoidable waste in research – was prompted by experience with JLA research Priority Setting Partnerships. In a paper published in *The Lancet* in 2009, Iain Chalmers and Paul Glasziou (2009) [5] estimated that over 85% of the investment in biomedical research was being avoidably wasted. The paper led *The Lancet* to invite Chalmers and Glasziou to coordinate the preparation of a series of papers with over 40 co-authors (Macleod et al. 2014) [6] dealing with five important sources of research waste: waste in deciding what research to fund (Chalmers et al. 2014) [7]; inappropriate research design, methods, and analysis (Ioannidis et al. 2014) [8]; inefficient research regulation and management (Salman et al. 2014) [9]; inaccessible research information (Chan et al. 2014) [10]; and biased and unusable research reports (Glasziou et al. 2014) [11]. These papers set out some of the most pressing issues, recommended how to increase value and reduce waste in biomedical research, and proposed metrics for stakeholders to monitor the implementation of these recommendations. Since the Lancet series was published in early 2014 there has been encouraging evidence of steps being taken to reduce waste, particularly by research funders (Glasziou and Chalmers 2018) [12].

The present article describes a third strand of work which has existed throughout the life of the JLI between 2003 and 2018, namely, to provide books, websites, databases and talks for the public and professionals about why fair tests of treatments are essential, what the features of fair tests are, and how everyone can play their part in promoting critical thinking and better research for better healthcare.

## Books

### *Testing Treatments*, 1st edition

At the end of 2002, Imogen Evans, a physician then working at the MRC, was considering writing a book for the public about clinical trials. She invited Iain Chalmers to co-author the book and the JLI to coordinate its preparation. At the suggestion of Mike Clarke, then director of the UK Cochrane Centre, Hazel Thornton, a breast cancer patient who had co-founded a Consumers Advisory Group for Clinical Trials, was invited to become a third co-author.

*Testing Treatments: better research for better health care,* authored by Imogen Evans, Hazel Thornton and Iain Chalmers, was published by the British Library in 2006 [13]. The 100-page book was written for anyone who wanted to understand better why treatments need to be tested rigorously; how treatments can be tested fairly; and how everyone interested in health care has a role to play in promoting better research for better health care.

The book’s eight chapters reflected these considerations. Chapter 3 – *Key Concepts in fair tests of treatments* – addressed methodological issues relevant to the fair testing of treatments:

‘Why comparisons are essential’;

‘Why comparisons must address genuine uncertainties’;

‘Avoiding biased comparisons’ (from differences in patients being compared and the ways treatment outcomes have been assessed); and

‘How to interpret unbiased comparisons’, taking account of the play of chance and all the relevant evidence.

Translations of the first edition of *Testing Treatments* were published in Arabic, Chinese, German, Italian, Polish and Spanish. Texts of all these versions were made freely available through the James Lind Library (www.jameslindlibrary.org), a website illustrating the evolution of fair tests of treatments (Chalmers et al. 2008 [14]; Chalmers 2015 [15]; and see below). The website had been launched at the Royal College of Physicians of Edinburgh in 2003 (250 years after Lind’s treatise on scurvy), and incorporated as an element of the JLI’s programme of work the same year.

### *Testing Treatments*, 2nd edition

The reception of the first edition of *Testing Treatments* prompted plans for an enlarged (200-page) second edition. The 16 pages devoted to Key Concepts in fair tests of treatments in the first edition was expanded to 41 pages across three chapters in the second edition. The second edition of the book also dealt with some of the themes that had been missing from the first edition. These included, for example, overtreatment, screening, research regulation, and the use of research evidence to inform decisions in health care.

The co-authors of the first edition of the book were pleased that Paul Glasziou, an Australian general practitioner, joined as a fourth co-author to prepare the 2nd edition of the book, and to add explanatory diagrams. Comments were solicited on drafts of this 2nd edition from a hundred people, including many lay people as well as health professionals, journalists, and researchers.

The book was published by Pinter and Martin in 2011 [16]. By the end of 2018, translations were available in Arabic, Basque, Catalan, Chinese, Croatian, Danish, Farsi, French, German, Italian, Japanese, Korean, Malay, Norwegian, Polish, Portuguese, Russian, Spanish, Swedish, Thai, and Turkish. Texts of the 2nd edition of *Testing Treatments* and translations are available free both through the Testing Treatments sibling websites www.testingtreatments.org and through the Cochrane Collaboration’s learning programmes http://training.cochrane.org/search/site/testing-treatments. The book is being downloaded for free around 100 times a month, and 5965 published copies have been sold. Audio versions of the book in Chinese, English and Spanish are also freely available on the relevant websites. The audiobook in English has accrued over 4000 listens on Sound Cloud.

*Testing Treatments* has been well received. In his Foreword to the 1st edition, Nick Ross, a broadcaster, wrote that the book is “good for our health [and] important for anyone concerned about their own or their family’s health, or the politics of health.” In his Foreword to the 2nd edition Ben Goldacre, a researcher and science writer, wrote “I genuinely, truly, cannot recommend this awesome book highly enough for its clarity, depth, and humanity.”

*Testing Treatments* has also been well received by others who have published reviews of the book. Its strengths were summed up succinctly by a Norwegian physician who judged the book to be “Important, scary, and encouraging”. Others have written that *Testing Treatments* is “a terrific little book” (*BMJ*); “a timely, inspiring read” (*Br J Gen Pract*); “… the best available introduction to the methods, uses and value of fair testing” (*Health Affairs*); and that it “… will inform patients, clinicians and researchers alike” (*Lancet*), and “should be required reading for everyone interested in healthcare” (*J Clin Res Best Practices*). The European Writers’ Association noted that the book “… encourages users and providers of healthcare to question assumptions, detect biases, and raise questions about the quality of the evidence if they find it unconvincing”, and the Patient Information Forum concluded that “patient groups would do well to read, inwardly digest and then spread the word” (https://en.testingtreatments.org/book/the-book/reviews/).

Perhaps the best endorsement of the enduring value of *Testing Treatments* is that people continue to go to the trouble of translating it into other languages. These reactions to the book probably reflect extensive pre-publication piloting of the text with lay and professional readers.

**Fig. 1:**
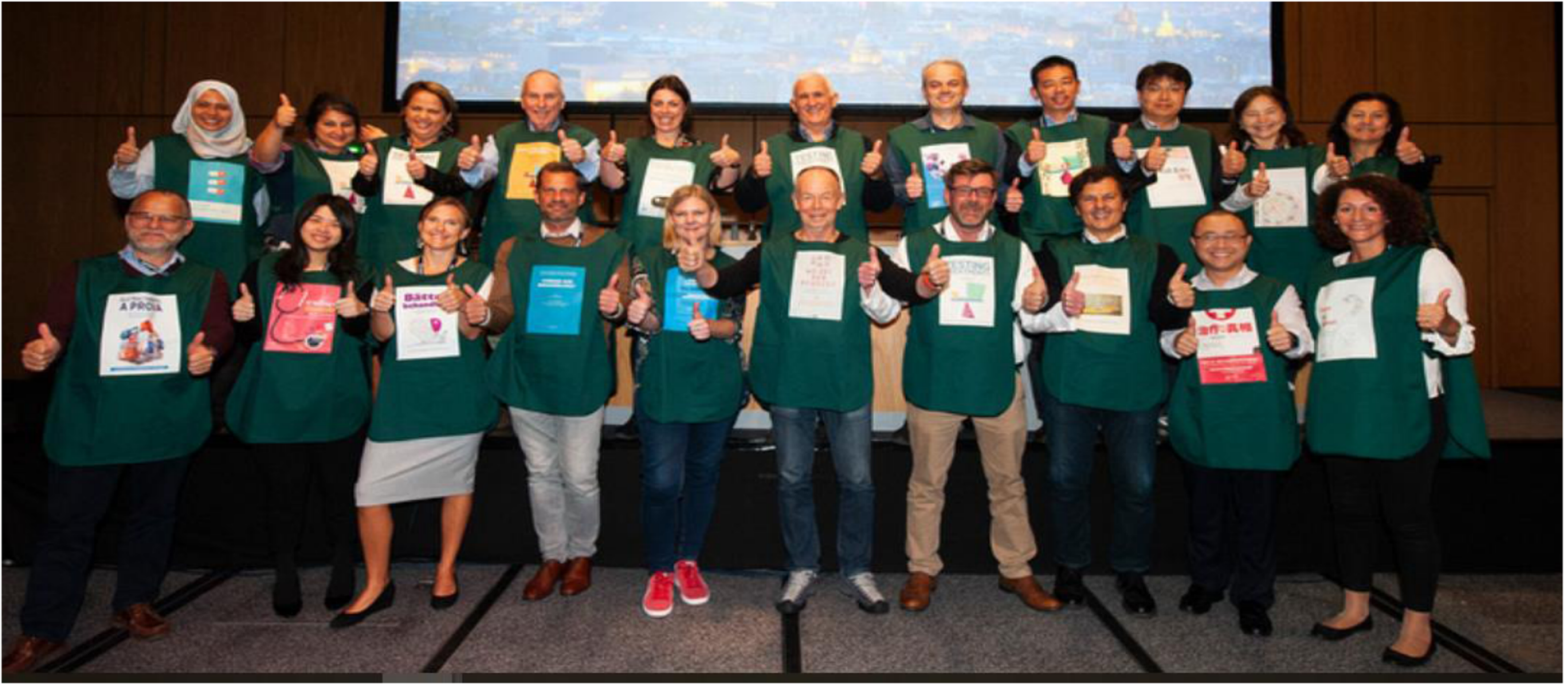
The Testing Treatments international Editorial Alliance, 18 September 2018, Cochrane Colloquium, Edinburgh. *Back Row*: Siti Nurkamilla (Malay), Mona Nasser (Farsi), Karla Soares-Weiser (Portuguese), Iain Chalmers (representing Turkish), Marimar Ubeda (Basque), Andy Oxman (English), Giordano Perez Gaxiola (Spanish), Rintaro Mori (Japanese), Myeong Soo Lee (Korean), Liliya Ziganshina (Russian), Mona Nabulsi (Arabic). *Front Row*: Gerard Urrutia (Catalan), Benjarin Santatiwongchai (Thai), Minna Johansson (Swedish), Karsten Jorgensen (Danish), Astrid Austvoll-Dahlgren (Norwegian), Gerd Antes (German), Douglas Badenoch (English), Roberto D’Amico (Italian), Yaolong Chen (Chinese), Tina Poklepovic Pericic (Croatian). *Missing*: Philippe Ravaud (French), Metin Gulmezoglu (Turkish)

A TTi Editorial Alliance was convened by Iain Chalmers in 2013 to share experiences of using translations of the book in different languages (Chen et al. 2015) [17]. Yaolong Chen, of Langzhou University, took over the convenorship of the TTi Editorial Alliance at the end of 2018 (Fig. 1). Because of the internationalisation of the TTi, we have chosen to refer to TTi as Testing Treatments international.

At the time of writing, in December 2018, there are no firm plans for a 3rd edition of *Testing Treatments*. However, Paul Glasziou has had exploratory discussions about extending coverage beyond tests of treatments to encompass tests of (diagnostic and screening) tests.

### James Lind's Introduction to Fair Tests of Treatments, 2019

The 2nd edition of *Testing Treatments* is nearly 200 pages long, and there have been occasional suggestions that a shorter book covering similar ground would be welcomed. As appropriate texts and images in six languages are already available as explanatory essays in the James Lind Library (www.jameslindlibrary.org, and see below), these will be compiled and published in a short book in 2019. Although the book should be valuable in its own right, we hope it will also help to draw attention to The James Lind Library.

## Websites

### *The James Lind Library* (www.jameslindlibrary.org)

The web-based James Lind Library (JLL) was created in 2003, in collaboration with the Sibbald Library at the Royal College of Physicians of Edinburgh, to explain and illustrate the development of fair tests of treatments in health care. The website contains three main types of material, categorized by methodological topic (www.jameslindlibrary.org/topics/). As of the end of December 2018, 22 *explanatory essays* (mentioned above) provide an overview in six languages of the topics addressed in the JLL. There are well over *1000 records* represented in the JLL, including scans of key passages at a minimum (with the majority of these sourced from the Sibbald Library), and often including links to full text, portraits, and other material.

The third element of the JLL comprises over 250 original articles. These JLL articles provide brief histories, commentaries, biographies, personal reflections, and summaries and full texts of relevant doctoral theses. In the year of the JLL’s launch, Kamran Abbasi, editor of the *Journal of the Royal Society of Medicine* (*JRSM*) suggested that JLL Articles could be republished in each monthly issue of the *JRSM*. This republication arrangement was initiated with an article by Richard Doll on the introduction of clinical trials using factorial designs in the late 1940s and early 1950s to compare more than two treatments concurrently in the same study. More than 200 articles have since been republished in the *JRSM*, allowing them to be indexed in PubMed and other bibliographic databases. The readership of these JLL articles is further extended through weekly posts on Twitter and Facebook.

In recent years, under the Associate Editorial responsibility of Douglas Badenoch assisted by Paul Glasziou, the focus of the JLL has broadened beyond its previous emphasis on the history of the control of systematic errors *(biases*) and random errors (*the play of chance*) to include material illustrating innovations in how research evidence can serve the interests of patients more effectively. For example, records and articles are being added to illustrate key milestones and developments in evidence summaries, levels of evidence, and evidence appraisal.

In 2015, the Centre for Evidence-Based Medicine (CEBM) at Oxford University hosted the launch of a beautiful redesign of the JLL website created by Douglas Badenoch, Robin Layfield and their colleagues. More recently, JLL materials have also been used in the development of the CEBM’s Catalogue of Bias (www.catalogofbias.org).

The JLL is an internationally useful resource, for a wide range of users. It receives around 200 visits per day, with an aggregate total of more than 120,000 page views per year. In a survey in January to April 2018, more than 400 users of the JLL completed a short, single-question survey. This revealed that just over half the users are students; 14% identified themselves as health professionals, 14% as researchers, 4% as members of the public, 4% as teachers, and 2% as librarians or information specialists.

The principal editorial responsibility for The James Lind Library was with Iain Chalmers’ until the end of 2018, when Mike Clarke took over from him, with support from Patricia Atkinson and Douglas Badenoch.

### *Testing Treatments international (TTi) English* (www.en.testingtreatments.org)

**Fig. 2:**
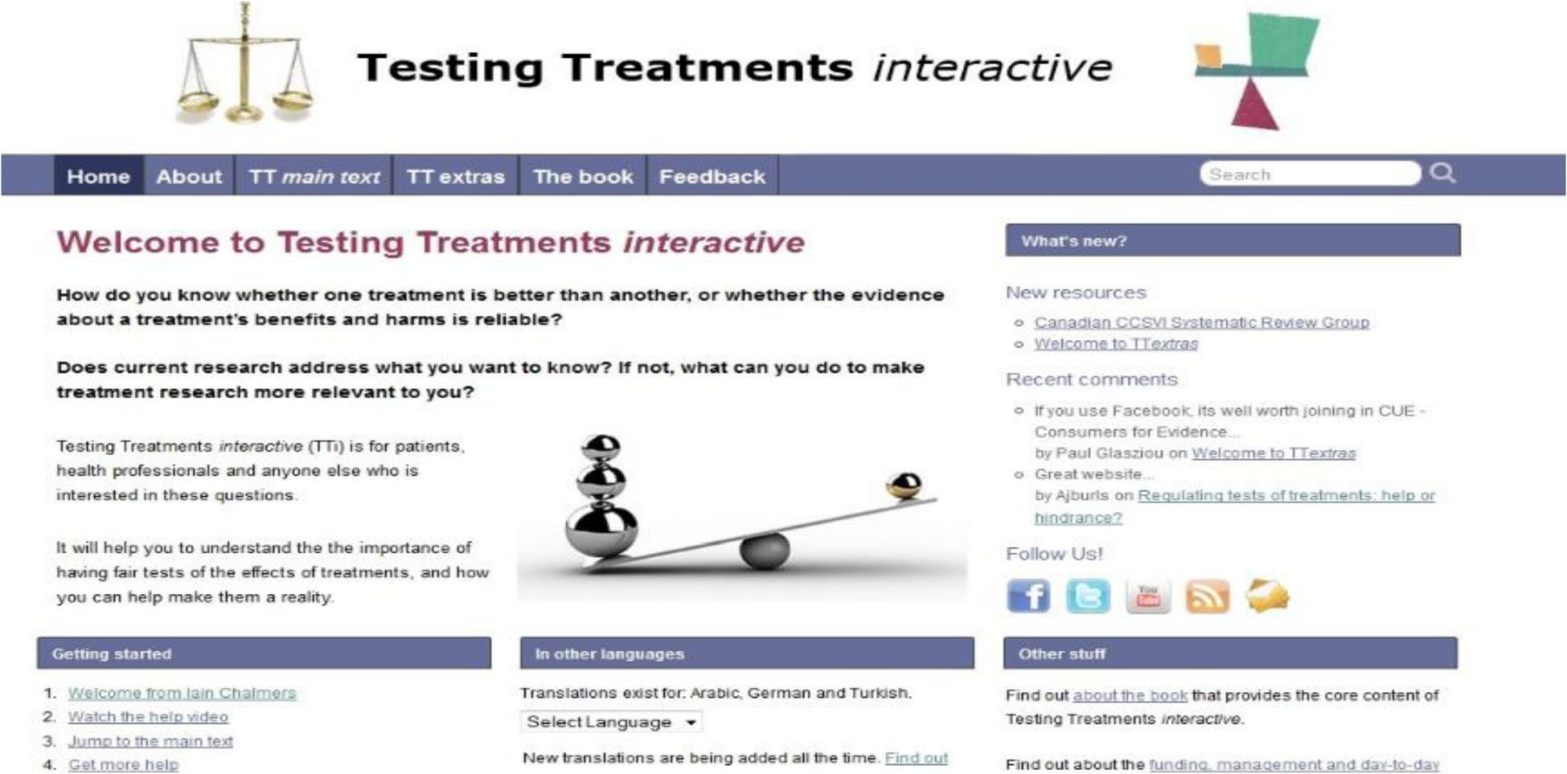
Screenshot of TTi English homepage, 2012

Release of the 2nd edition of *Testing Treatments* (Evans et al. 2011) [16] was accompanied by the launch of a website entitled Testing Treatments *interactive* (TTi) Fig.2. The website was created principally to make the text of the book freely and widely available. Translators of the book into twenty other languages have followed our example by establishing ‘sibling’ TTi websites to host translations of the book in their different languages. In addition to hosting texts of the book, TTi English and some other TTi websites added other resources to their websites. TTi English, for example, developed and hosted the Critical thinking and Appraisal Resource Library (CARL) (Castle et al. 2017) [18], and it hosted the Key Concepts database, the plain language glossary (GET-IT), the Claim Evaluation Tools (see below), and a guide to reliable information about the effects of treatments (https://en.testingtreatments.org/create-test-claim-evaluation-tools-database/).

Throughout the development of the TTi English website we have consulted lay and professional end-users to find out what they think of our work. The first formal assessment of the site was conducted internally in 2012–13. In late 2012, we surveyed users of the website to find out more about them and their reasons for using the site. We found that most users were “intermediaries”, that is, people who were involved professionally in communicating or teaching about health research. Following on from this, we recruited 20 such “intermediaries” for hands-on user testing and semi-structured interviews. They helped us to identify some important barriers to user understanding.

In particular, many users, particularly patients, expected a website called “Testing Treatments” to tell them about specific treatments, rather than the process of evaluating the effects of treatments in general. They also told us that we needed to make our credentials clearer (to reassure users we were not a “crank site”), and to provide a glossary of jargon terms.

In response, we redesigned the site to guide users to sources of information about specific treatments (https://en.testingtreatments.org/testing-treatments-interactive-tti/sources-trustworthy-information-treatment-effects/), and the JLI made clear how our work was funded, and introduced the personnel behind the project. We also collaborated with colleagues in Scotland and Norway in work funded by the European Commission to develop a plain language glossary – GET-IT (Moberg et al. 2018) [19]. This database was designed by Robin Layfield and Douglas Badenoch, who remain responsible for maintaining and developing it.

**Fig. 3:**
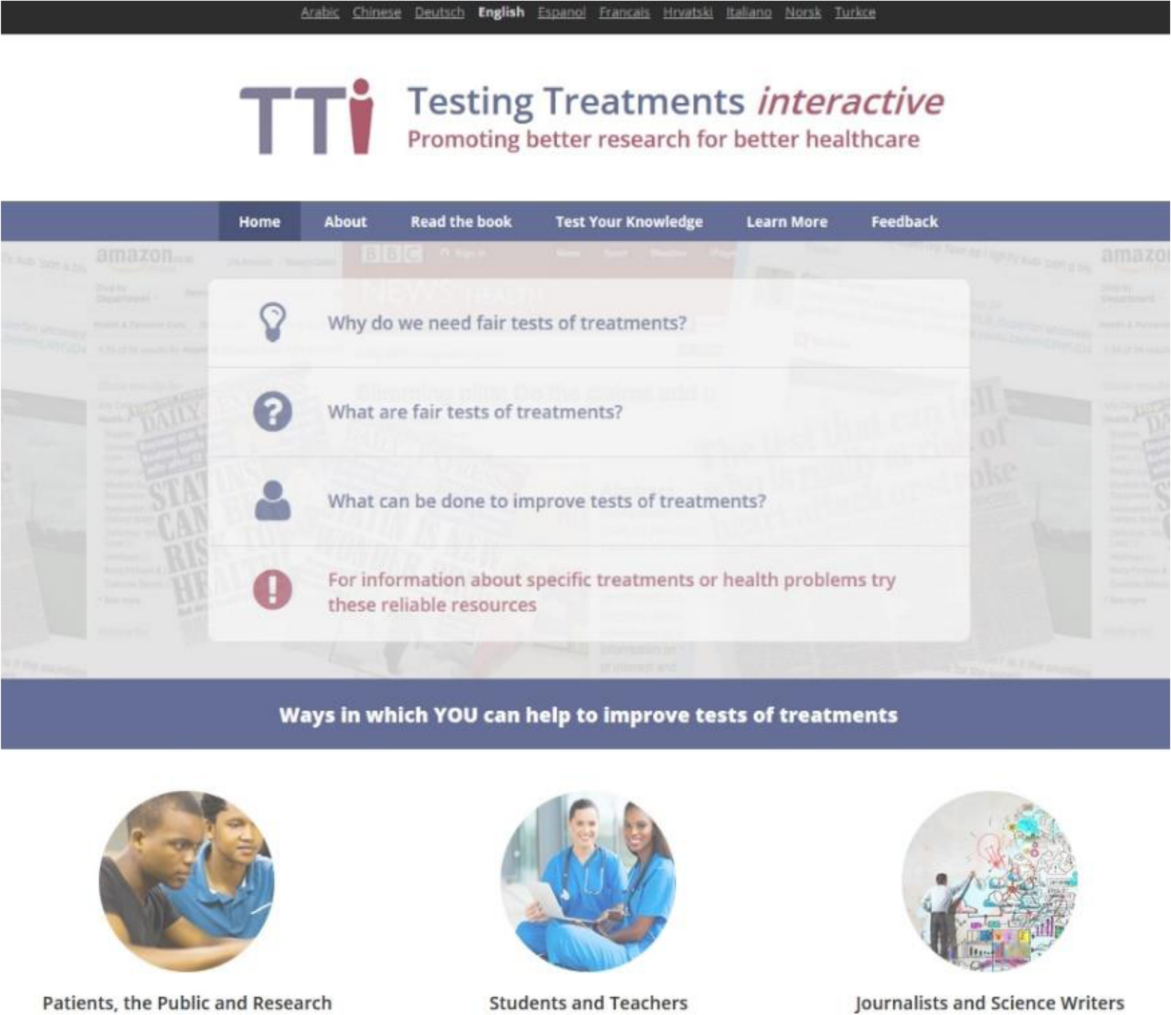
Screenshot of TTi English homepage, 2014

In 2014, we commissioned the Critical Appraisal Skills Programme (CASP) to do an external assessment of TTi English based on interviewing and testing lay people and some professionals. The further development of the website reflected the findings. We made clear that the users we were targeting were ‘intermediaries’ - teachers of children and adults, journalists, and science writers – although not explicitly excluding lay users who wished to teach themselves. We simplified the home page, to emphasise that the website is concerned with ‘healthy scepticism’; to give more emphasis to the resources contained in the site; and to reduce clutter on the home page Fig. 3.

This simplified front-end was developed in parallel with the Informed Health Choices Project’s creation of a Key Concepts framework for evaluating treatment claims (Austvoll-Dahlgren et al. 2015 [20]; Chalmers et al. 2018 [21]; Oxman et al. 2018 [22]; and see below).

TTi English content was re-indexed using this taxonomy, and new resources were added regularly to encourage return visits. By this time, we had built up a substantial collection of learning resources (TT Extras), which provided a clearer offering to users.

**Fig. 4:**
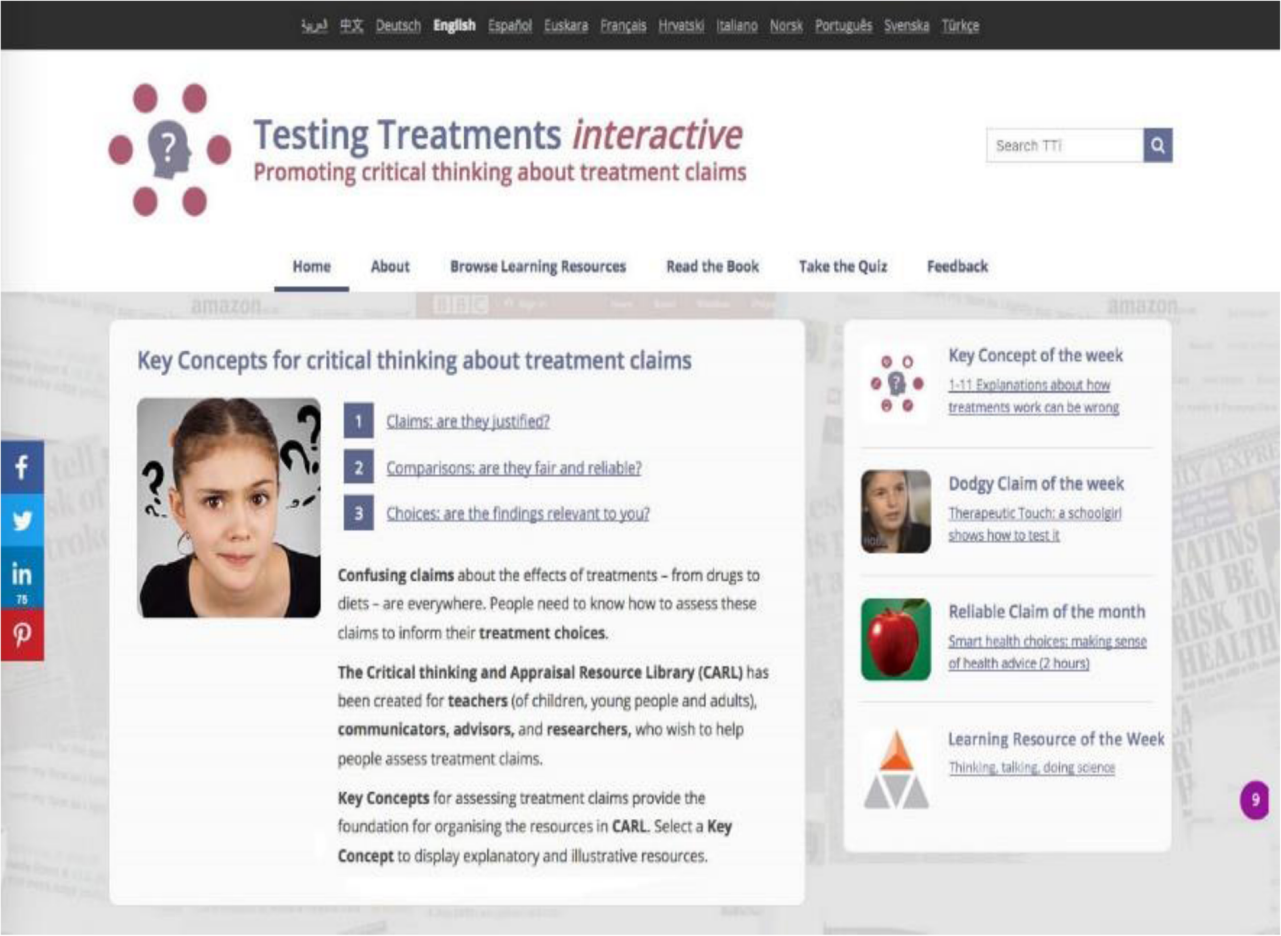
Screenshot of TTi English homepage, 2016

This content was expanded into the Critical thinking and Appraisal Resource Library (CARL). Open access learning resources were identified to supplement the learning from the book. These were were collated and indexed using the Key Concepts taxonomy. The resulting database was embedded in the TTi English website (Fig. 4). The methods used to create CARL have been documented by Castle and his colleagues (2017) [18].

A further external assessment was commissioned in 2016 to assess these changes. The findings this time made clear that we had over-reached ourselves! Feedback made clear that the website was attempting to serve the needs of too many diverse groups, with the result that people coming to it were unclear for whom it had been developed. We concluded that we had been too ambitious in imagining that the website could serve the needs of teachers of too wide a range of learners - primary school children, teenagers, undergraduates, graduates, and individual members of the public. It was clear that a radical rethink was required.

**Fig. 5:**
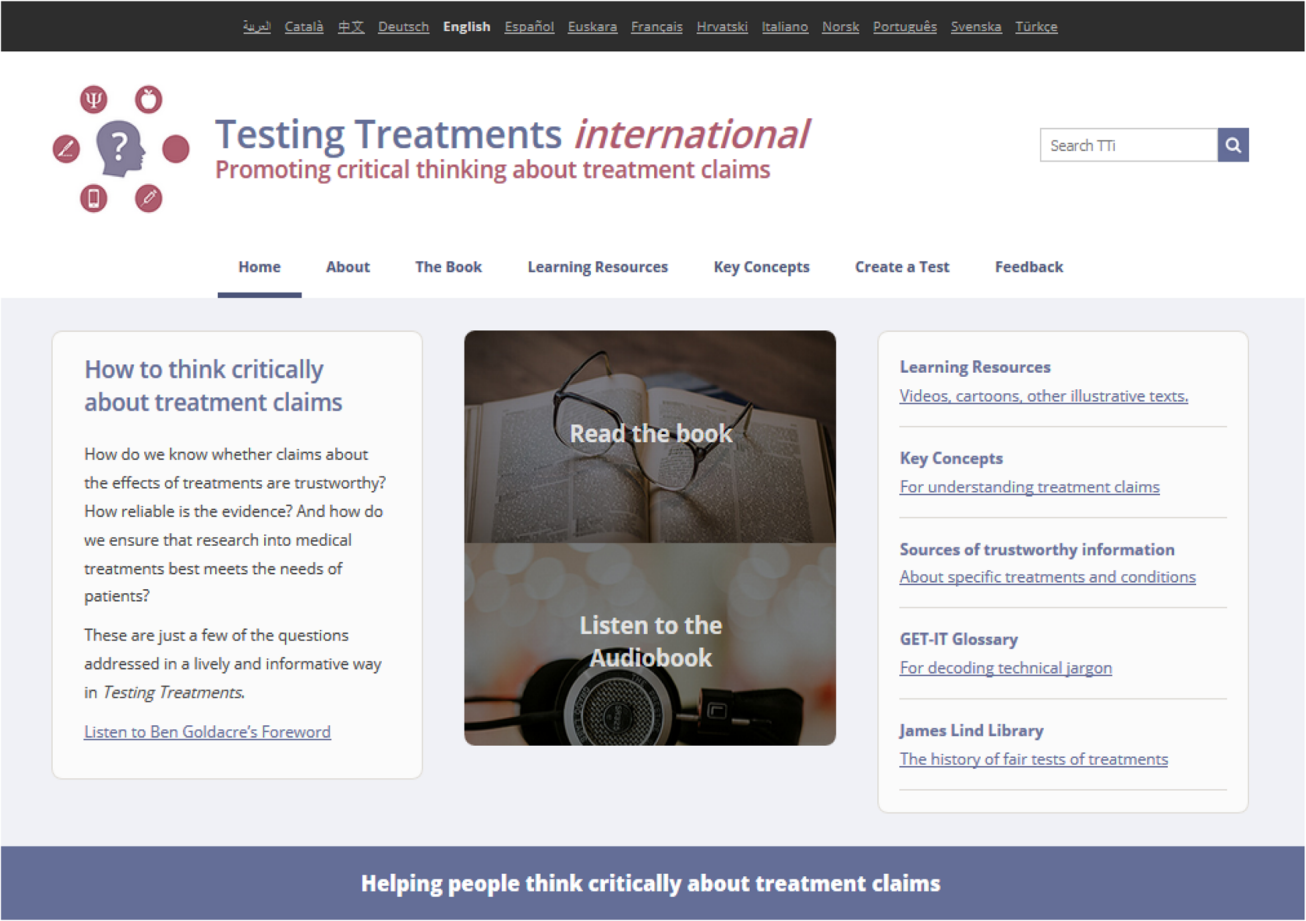
Screenshot of TTi English homepage, 2018

After consultation and discussion by the editorial team, we decided that two distinct websites should be created from the TTi English site. One of these would return to an earlier, simpler design with the public in mind; the other would be developed explicitly to meet the needs of Teachers of Evidence-Based Health Care Fig. 5.

## Website of resources for teachers of evidence-based health care

In 2018, the ideas and methods of the CARL database were extended to include materials for teaching EBHC to undergraduate or postgraduate health professionals, and students with a special interest in EBHC, such as Students for Best Evidence [https://www.students4bestevidence.net/].

The concept of such a database and a prototype website were discussed over two meetings at the Evidence-Based Health Care Conference in Sicily, setting out the scope and processes. As the design and conception developed, the group provided feedback, and helped to refine functionality and design, and source further content. The shared aim is for a global sharing platform of materials for teaching EBHC, with an emphasis on those that have reliable evidence of effectiveness.

For inclusion, learning resources must be freely available and be relevant to one or more of: the EBM Stages (0 Why EBM? 1 Asking focused questions, 2 Finding evidence, 3 Appraising evidence, 4 Decision making, 5 Evaluating performance), or one of the Key Concepts.

The development of the new website was supported by the JLI until its launch on 1 November 2018. Support to maintain the new website is now provided by the National Institute for Health Research (NIHR) and Cochrane UK, and the International Society for Evidence-Based Health Care (EBHC).

Given the diversity of courses, stages, and curriculum contexts, the database provides modular materials for a variety of uses such as: (i) preparing a lecture at short notice, (ii) needing to find an amusing or engaging illustration of EBHC concepts, (iii) planning a course on EBHC, (iv) finding preparatory reading for students, or (v) first-time course development.

The database was seeded with over 550 resources identified through systematic searching of the internet (Castle et al. 2017) [18]. The network started with about 40 members from around the world recruited at the Sicily meeting.

**Fig. 6:**
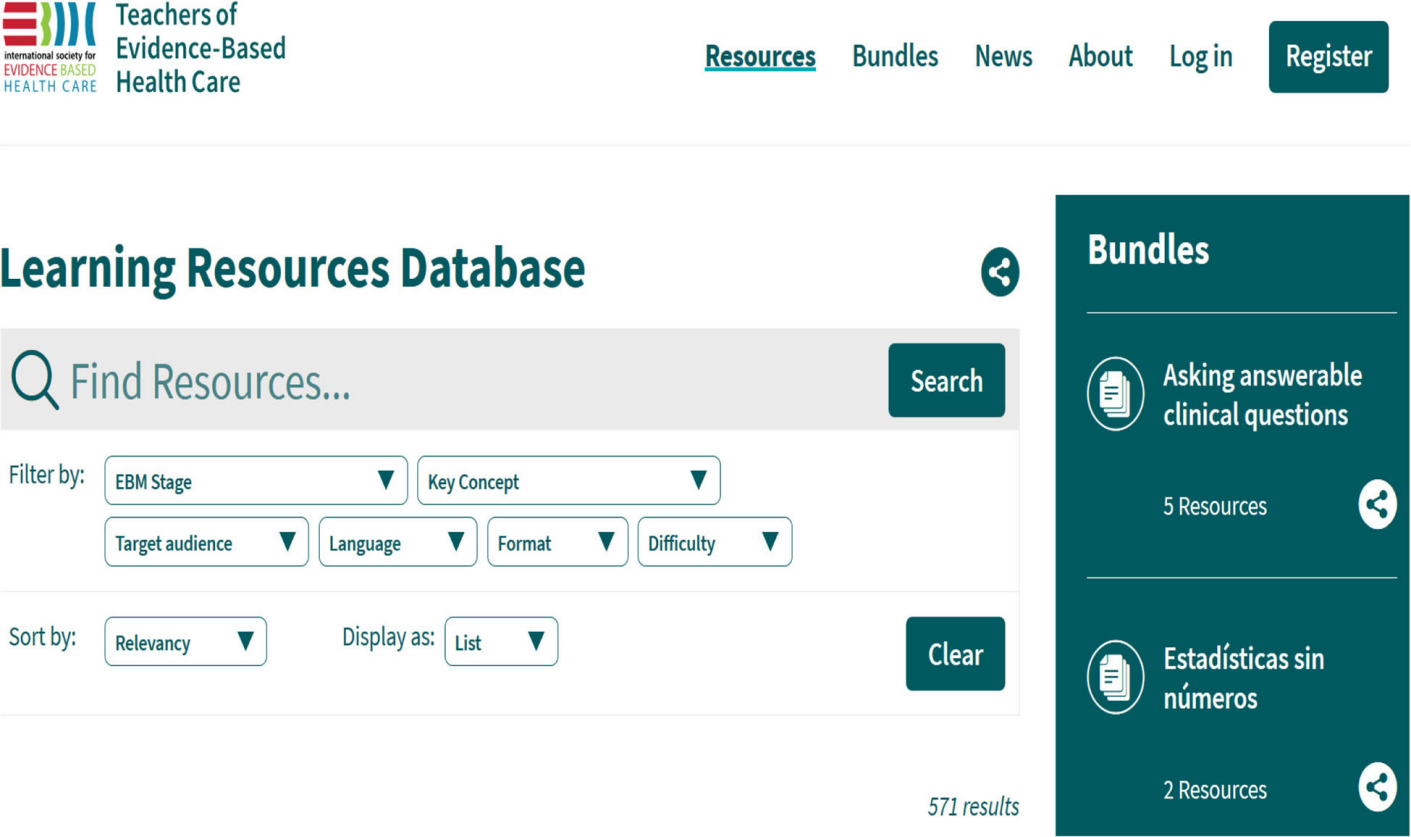
Teachers of EBHC Learning Resources Database, 2018

To be included in CARL, a learning resource must be freely available and relevant to one or more of the: EBM Stages, EBHC Competences, or Key Concepts, as set out by Loai AlBarqouni and his colleagues (2018) [23]. Content is edited by Patricia Atkinson, Douglas Badenoch and Paul Glasziou, with support from an Editorial Board of six. End users suggest content for the Database, which is checked, indexed and approved by editors before publication in the Database. Users can sign up for a regular email digest of new resources that have been added to the Database. Members can comment on or rate resources and create ‘Bundles’ of resources for their own use Fig. 6.

The Teaching EBHC website http://www.teachingebhc.org) was launched at the annual conference of the International Society for Evidence-Based Health Care in the United Arab Emirates on 1 November 2018. Since then, over 150 additional members have registered on the website, and are contributing new learning resources.

Paul Glasziou will be the caretaking editor of TTi English from April 2019, and he coordinates the development of the website for Teachers of Evidence-Based Healthcare. Douglas Badenoch remains intimately involved in both websites.

## GenerationR website (http://generationr.org.uk/)

The Young Persons’ Advisory Groups (YPAGs) for clinical trials launched GenerationR at the Science Museum in London in September 2013 to tell others about how they were helping researchers design clinical trials involving children. They invited Iain Chalmers to contribute to the launch and asked him to speak about waste in research and how they might help to reduce it. This led to discussions with representatives and facilitators of all five YPAGs at many meetings during 2014 and 2015.

**Fig. 7:**
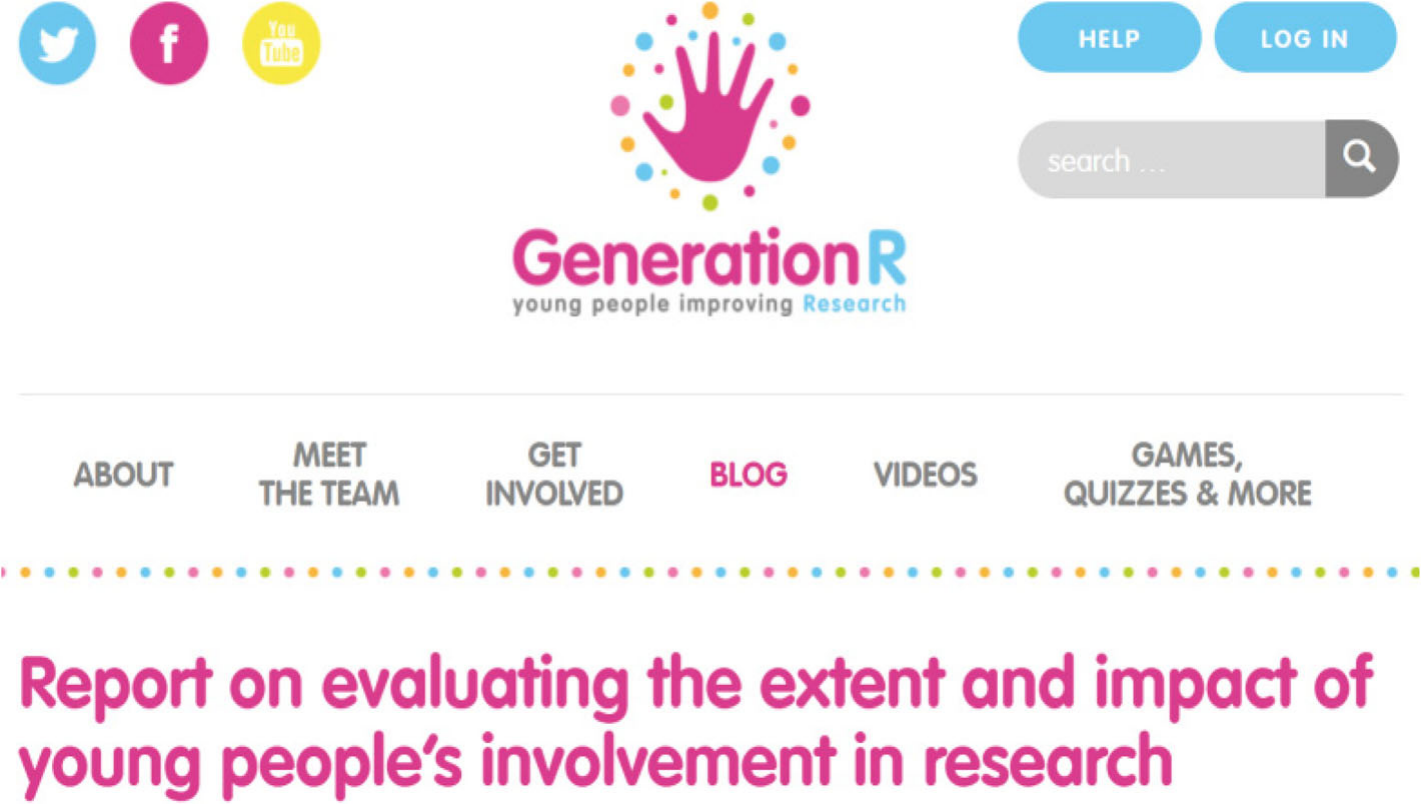
Screenshot of GenerationR homepage

The GenerationR report published in the spring of 2014 called for the creation of a dedicated GenerationR website. This was achieved with funds provided through the JLI and five planning meetings with YPAG members coordinated by Jennifer Preston (Liverpool YPAG), and facilitated by Douglas Badenoch, with support from Patricia Atkinson and Iain Chalmers. The website was launched in 2014, initially within TTi English, then separately at http://generationr.org.uk/ Fig. 7.

With the website established, the JLI focused on one of the other recommendations in the GenerationR report, which asked for “Work with the education sector to promote clinical research education in schools, sharing resources such as Testing Treatments interactive …”. Iain Chalmers tried to promote collaboration among YPAGs to develop and evaluate training materials, with a view to creating a cadre of young people equipped to promote learning in schools (‘GenR Ambassadors’). This effort was unsuccessful, however, not least because YPAG facilitators were victims of yet another internal reorganisation of the NHS, with inevitable uncertainties about jobs and lines of accountability. These developments were a disappointing blow to JLI’s hopes to develop a meaningful GenerationR/JLI collaboration. Iain Chalmers decided that his time and other JLI resources should be redeployed instead through involvement with primary schools in the work of the Informed Health Choices Project (see below).

## Databases

Since its inception in 2011, TTi English has hosted and made accessible several ‘proto-databases’. As the JLI came to an end, these have been established as stand-alone databases which can be plugged into a variety of websites.

## Critical thinking and assessment resource library (CARL)

In April 2011, the JLI hosted an international meeting on ‘Enhancing Public Understanding of Health Research’, held at Kellogg College, Oxford. The meeting was supported by the National Institute for Health Research (NIHR) and the Wellcome Trust. In preparation for the meeting, an inventory of interactive applications and learning materials to teach people how to be critical users of information about the effects of health care was prepared.

The meeting led to the creation of an international Network to Support Understanding of Health Research (NSUHR) and a proposal to develop *BS Detective –* a social media project to help people distinguish trustworthy from untrustworthy health research and reporting. Extension and maintenance of the inventory of learning resources was envisaged as a component of this initiative. We were disappointed that, despite encouragement from and detailed consultation with advisers at the Wellcome Trust, the proposal for *BS Detective* was rejected by the Trust.

Fortunately, NIHR’s support for the JLI enabled extension of the initial inventory of learning resources prepared for the 2011 conference. To supplement the text of the *Testing Treatments* book, learning resources in the forms of text, video, audio and other media were added as ‘TT Extras’ to the TTi English website.

In 2012, funds were obtained from the European Commission “to identify and develop materials to enhance public understanding of clinical trials”. The JLI was one of the partners in ECRAN - the European Communication on Research Awareness Needs (Mosconi et al. 2016) [24]. The JLI was a partner in the ECRAN project and learning resources identified and developed by ECRAN were incorporated as TT Extras in the TTi English website.

The TT Extras provided the foundation for developing the Critical thinking and Appraisal Resource Library (CARL) to help people understand and apply key concepts in assessing treatment claims (Castle et al. 2017) [18]. Additional learning resources were identified through relevant systematic reviews (Nordheim et al. 2016 [25]; Austvoll-Dahlgren et al. 2016 [26]; Cusack et al. 2018 [27]; Albarqouni et al. 2018 [23]) and online and database searches.

CARL currently contains over 550 open-access learning resources in a variety of formats: text (including extracts from *Testing Treatments* and explanatory essays from the James Lind Library), audio, video, web pages, cartoons and lesson materials (Castle et al. 2017) [18]. At the end of 2018, responsibility for the maintenance and development of CARL was transferred from the JLI to the Society of Evidence-Based Health Care.

## Informed health choices databases

Soon after publication of the 2nd edition of *Testing Treatments* in 2011, Andy Oxman, then a researcher at the Global Health Unit in the Norwegian Knowledge Centre for the Health Services, observed that the *Testing Treatments* book alluded to many of the Key Concepts that people need to consider when assessing the trustworthiness of claims about the effects of treatments. He proposed assessing whether these Key Concepts could be taught to and successfully applied by primary school children in low income countries when they were assessing the trustworthiness of claims about health.

A project proposal – ‘Supporting informed healthcare choices in low-income countries’ - was developed and submitted to the GLOBVAC programme of the Research Council of Norway. The project came to be known as the Informed Health Choices (IHC) Project (www.informedhealthchoices.org). Iain Chalmers was grateful to have been invited to become a member of the substantial project team, along with other members in Norway and East Africa, and to involve the JLI in the project.

Iain Chalmers’ principal involvement has been by working with Andy Oxman and Astrid Austvoll-Dahlgren to (i) identify the Key Concepts to be taught to learners, and (ii) develop multiple-choice questions - the Claim Evaluation Tools - to assess people’s ability to apply the Key Concepts in practice (Austvoll-Dahlgren et al. 2016 [25]; 2017 [28]). Since its creation, information about the Key Concepts and Claim Evaluation Tools has been made available through TTi English but separate databases have been or are being created for these resources.

### Key concepts database

A first list of 32 Key Concepts relevant to assessing the trustworthiness of treatment claims was published by Astrid Austvoll-Dahlgren and colleagues in 2015 (Austvoll-Dahlgren et al. 2015) [20]. Since its first iteration, the list has been reassessed every year, and currently consists of 44 concepts (Chalmers et al. 2018 [21]; Oxman et al. 2018 [22]). The list has provided an invaluable basis for organising and coding materials in TTi English, the JLL, and CARL. The Key Concepts have also been used by Students 4 Best Evidence (coordinated by Cochrane UK), which has published blogs and short videos on all of them (https://www.students4bestevidence.net/tag/keyconcepts/).

The list of Key Concepts serves as a syllabus for developing learning resources. As such, it is a starting point for teachers or researchers to develop tailored interventions to help people in specific target groups to make informed health choices. Although the list of IHC Key Concepts was developed to promote informed choice of interventions to protect health, it has been shown to be applicable in education more generally (Chalmers et al. 2018 [21]; Sharples et al. 2017 [29]).

The draft 2018 edition of the Key Concepts for promoting informed choices in health was presented and discussed at international meetings in June in Oxford and in Edinburgh in September. A meeting in December 2018, convened by Andy Oxman and Iain Chalmers, explored the extent to which the Key Concepts can be applied to interventions in other areas in which claims are made about the effects of interventions – specifically, about agricultural, economic, educational, environmental, informal learning, international development, management, nutritional, planetary health, policing, social welfare, speech and language, and veterinary interventions.

### Claim evaluation tools database

All the questions with the IHC’s Claim Evaluation Tools database have been developed for use in children (from the age of 10) as well as for adults (including health professionals). These multiple-choice questions can be used:

- to test critical abilities in school and other teaching settings
- to evaluate outcomes of educational interventions assessed in randomised trials
- to gauge critical abilities in a population, and thus provide background information to help tailor interventions to address people’s educational needs.

The multiple-choice questions are currently available in English, Norwegian, Chinese, Spanish, German and Luganda, and a network of people working with or wishing to work with the tools is emerging.

## Evaluating the effects of educational interventions

Although feedback on the resources discussed so far in this article has been encouraging, there is no hard evidence that they have promoted critical thinking about treatment claims, let alone whether they have been useful in applying any learning in practice when making health choices. As made clear in a systematic review by Cusack and her colleagues (2018) [27], robust evaluation of well-intentioned resources designed to equip people to be ‘health literate’ are vanishingly rare (see, for example, Woloshin et al. 2008; [30] https://www.ncbi.nlm.nih.gov/pubmed/23469386).

As mentioned above, the JLI failed to engage those responsible for the Young Persons Advisory Groups (YPAGs) and GenerationR in developing a programme to foster and assess relevant learning among the children and young people associated with the groups (Chalmers 2016) [31]. In the light of this disappointment, the involvement of Iain Chalmers, Patricia Atkinson, and the JLI in the IHC Project (www.informedhealthchoices.org) was very welcome, particularly as *Testing Treatments* had been the starting place for the IHC Key Concepts. The IHC Project developed teaching materials - a cartoon story book and a podcast – and used randomised trials involving over 10,000 Year 5 Ugandan primary school children (Nsangi et al. 2017) [32] and over 500 of their parents (Semakula et al. 2017) [33] to measure the effects of the teaching materials on their assessment of treatment claims. The teaching materials were successful in promoting application of the Key Concepts in judging the trustworthiness of treatment claims. A year later, the children’s abilities had continued to improve, but those of the parents had decayed (Nsangi et al.; Semakula et al. unpublished data).

These experiments have provoked interest around the world (http://www.informedhealthchoices.org/news/) and a 53-min BBC World documentary about the research was presented by David Spiegelhalter (The Documentary: You can handle the truth), who described the IHC Project as “a wholly new type of evaluative research.”

The JLI’s involvement in the IHC Project is the high point of the Initiative’s efforts to meet its objectives over the past 15 years.

## Conclusions

**Fig. 8:**
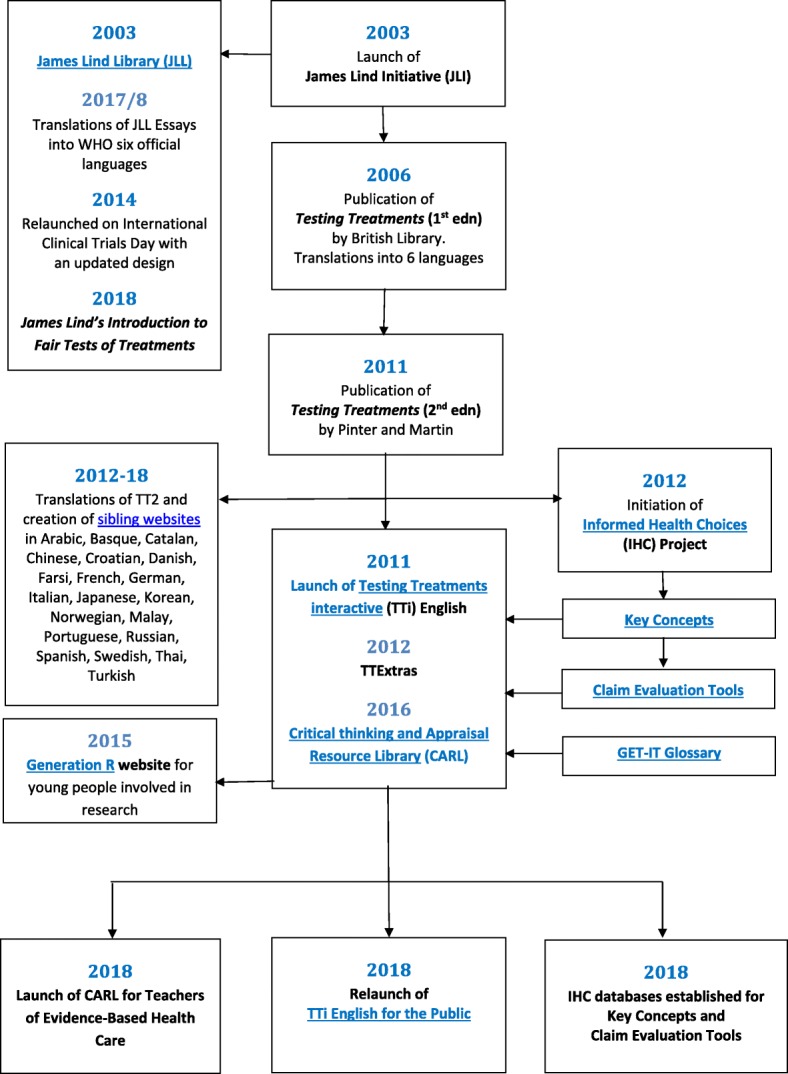
The evolution of Testing Treatments English and the James Lind Library, 2003–2018

This paper is the last of three papers summarizing the three principal strands of work of the James Lind Initiative during its 15-year life between 2003 and 2018. The first of these papers described the development of the James Lind Alliance (JLA), a process to help patients, carers and clinicians to agree on research priorities. The second paper reviewed efforts made by the JLI and others to expose and address avoidable waste in research. This third and final paper refers to the use of talks, seminars, books, websites, databases and controlled experiments to promote critical thinking about treatment claims. Figure 8 illustrates how – beginning in 2003 – the James Lind Library and the 1st edition of *Testing Treatments* evolved and led to the creation of several other teaching and learning resources, and to some important controlled trials to assess their effectiveness.

At the beginning of 2019, the JLI’s involvement with these resources ended with Iain Chalmers’ retirement. The contact authors for four of the principal resources are now as follows:

**James Lind Library:** Mike Clarke m.clarke@qub.ac.uk

**TTi English for the Public:** Paul Glasziou pglaszio@bond.edu.au

**Informed Health Choices:** Andy Oxman oxman@online.no

**CARL for Teachers of EBHC:** Douglas Badenoch douglas.badenoch@minervation.com

## Abbreviations

CARL: Critical thinking and Appraisal Resource Library
CASP: Critical Appraisal Skills Programme
CEBM: Centre for Evidence-Based Medicine
EBHC: Evidence-Based Health Care
ECRAN: European Communication on Research Awareness Needs
GET-IT: Plain language glossary
IHC: Informed Health Choices Project
JLA: James Lind Alliance
JLI: James Lind Initiative
JLL: James Lind Library
JRSM: Journal of the Royal Society of Medicine
MRC: (UK) Medical Research Council
NIHR: National Institute for Health Research
NSUHR: Network to Support Understanding of Health Research
TTi: Testing Treatments interactive/international
YPAGs: Young Persons’ Advisory Groups
